# Multi-omics characterization of RNF157 expression patterns in hepatocellular carcinoma and development of an RNF157-associated prognostic signature

**DOI:** 10.3389/fphar.2025.1738424

**Published:** 2026-01-22

**Authors:** Qingsong Yang, Meng Zhang, Chang-song Ma, Ao Li, Wenjun Zhang

**Affiliations:** Department of Hepatopancreatobiliary Surgery, Affiliated Chuzhou Hospital of Anhui Medical University (The First People’s Hospital of Chuzhou), Chuzhou, China

**Keywords:** hepatocellular carcinoma, multi-omics analysis, prognostic model, RNF157, tumor microenvironment

## Abstract

**Background:**

Hepatocellular carcinoma (HCC) remains a highly lethal malignancy due to tumor heterogeneity and treatment resistance. This study characterized E3 ubiquitin ligase RNF157 expression patterns in HCC through integrated multi-omics and single-cell analysis, developed an RNF157-associated prognostic signature, and explored its relationship with tumor microenvironment (TME) populations.

**Methods:**

Clinical and RNA expression data were obtained from TCGA, GEO databases, and scRNA-seq datasets (GSE149614). Protein-protein interaction networks were constructed via STRING database. Based on single-cell analysis revealing RNF157’s heterogeneous expression in cancer-associated fibroblasts (CAFs) and tumor-associated macrophages (TAMs), we performed validation experiments using lentiviral shRNAs targeting FAP in CAFs and CD11b in TAMs. All experiments included appropriate controls with three independent biological replicates.

**Results:**

Single-cell analysis identified significant heterogeneity in HCC samples, with RNF157 showing variable expression across cell types within the TME. The prognostic model demonstrated moderate predictive performance (AUC: 0.65–0.78 for 1–5 years survival). Flow cytometry confirmed successful experimental manipulation of TME populations, with reduced FAP + CAFs (45.54%→22.01%) and CD11b+ TAMs (35.03%→24.18%) following respective gene depletion.

**Conclusion:**

We characterized RNF157 expression patterns in HCC at single-cell resolution and established a prognostic signature with moderate predictive performance requiring independent validation before clinical application.

## 1 Introduction

Of all cancers impacting the world population, hepatocellular carcinoma (HCC) remains as a highly vexing neoplasm with significant public health implications. The survival rates at 5-year in HCC are still unacceptably low, with patient outcomes primarily driven by tumor heterogeneity, advanced stage disease upon diagnosis, and resistance mechanisms towards the available treatment options (Birol et al., 2024; Eggiman et al., 2024; Mulla et al., 2025; Zeng et al., 2024). Therefore, further understanding of the molecular mechanisms involved in HCC tumorigenesis, especially the exploration for key genes regulating tumor progression and therapeutic response is crucial to improve patient prognosis.

Among the large amount of genes exploring for their expression in HCC studies, RNF157 is a member of membrane anchor ring finger 1 family and has gained much attention because of its multifaceted role in tumor biology. As a member of the E3 ubiquitin ligase family, RNF157 is dysregulated in various cancer types and highly correlated with tumor genesis, progression, and prognosis. Although some studies have explored the role of RNF157 in cancer biology, its precise expression patterns within the tumor microenvironment and associations with different cell populations remain to be further elucidated (Guan et al., 2022; Xu et al., 2023).

The advent of multi-omics technologies has facilitated an integrative view into the molecular landscape of HCC based on multiple analytical layers including gene expression profiles, proteomic signatures and metabolomic landscapes. Besides, single-cell RNA sequencing technology has notably expanded our ability to discover the cellular heterogeneity of HCC and define different associated cell subpopulations with disease progression. These technological breakthroughs offer new possibilities for molecular stratification, prognosis estimation and personalized treatment in management of HCC (Li et al., 2022; Meng et al., 2023; Xu et al., 2025; Yu and Lei, 2023).

The aim of this research is to characterize the expression features of RNF157 in HCC by using multi-omics based approaches as well as constructing a prognostic signature associated with RNF157 expressions. Based on HCC series downloaded from publicly available databases, as well as bioinformatics tools and statistic methods, we screened out genes associated with prognosis of HCC and built a risk evaluation model. We performed Kaplan-Meier survival analysis and ROC curve analysis to investigate the discrimination ability of our model and aimed to identify potential molecular associations that may inform future mechanistic and clinical studies.

This study is expected to generate novel hypotheses and foundational data for precision medicine in HCC, inform future validation studies, and eventually provide a basis for improving the clinical outcome of HCC patients.

## 2 Methods

### 2.1 Data source

We obtained clinical data and RNA expression profiles for patients with hepatocellular carcinoma from the Cancer Genome Atlas (TCGA) and Gene Expression Omnibus (GEO) databases. There were also single-cell RNA sequencing (scRNA-seq) datasets of HCC samples downloaded from the GEO database (GSE149614). The GSE149614 dataset comprises samples from 10 HCC patients, with a total of 71,915 cells passing quality control filters. Clinical characteristics of these patients are summarized in Supplementary Table S1, including age, sex, tumor stage, etiology, and survival outcomes where available (Chen Y. et al., 2024; Chlahbi et al., 2024).

### 2.2 Study design and rationale for experimental validation

This study was designed as a bioinformatic discovery and characterization study to establish RNF157 expression patterns in HCC and develop an associated prognostic signature. The selection of FAP and CD11b as experimental targets was based on our single-cell RNA sequencing analysis, which revealed that RNF157 exhibited heterogeneous expression patterns across different cell types within the tumor microenvironment (TME), particularly showing significant associations with cancer-associated fibroblasts (CAFs) and tumor-associated macrophages (TAMs). FAP (Fibroblast Activation Protein) serves as a canonical marker for activated CAFs, while CD11b is a well-established marker for myeloid-derived cells including TAMs. The FAP and CD11b knockdown experiments were designed as proof-of-concept studies to demonstrate that the TME populations identified through our computational analyses as RNF157-associated could be experimentally modulated, rather than to directly test RNF157 function.

### 2.3 Identification of hub genes in core gene clusters

Venn diagram visualization was employed to identify overlapping genes implicated in disease processes, subsequently capturing core hub genes through intersection analysis of disease-associated gene sets. This approach facilitated efficient candidate gene selection for subsequent analysis. Utilizing the STRING database, we conducted protein-protein interaction (PPI) network analysis (Chen CM. et al., 2024; Du et al., 2024; Watanabe et al., 2024). This analytical framework enabled examination of interactions and relationships among intersected genes, providing mechanistic insights into potential disease processes.

### 2.4 Model building

Differential expression analysis was performed on genes demonstrating prognostic correlations, incorporating patient survival status and duration data. The TCGA-LIHC dataset was randomly divided into training (70%) and internal validation (30%) cohorts using a fixed random seed for reproducibility. We employed 10-fold cross-validation during model training to prevent overfitting. Additionally, we performed bootstrap resampling (1,000 iterations) to assess model stability and reported the confidence intervals for all performance metrics. External validation was performed using the GSE14520 dataset from the GEO database. To enhance analytical depth, unsupervised clustering methodologies were applied to stratify patients into distinct donor groups. Through this technique, we identified natural data clusters potentially representing discrete prognostic groups or disease subtypes. Following clustering procedures, we implemented a scoring framework designed to quantify prognostic implications of identified gene expression profiles. Principal component analysis (PCA) was conducted on the first two components to establish this analytical framework (Watanabe et al., 2024; Effati et al., 2024; Jeong et al., 2024; Rasul et al., 2024).

### 2.5 Single-cell level validation

The “Seurat” R package was used for scRNA-seq data analysis. Quality control of data was conducted after cells with a number of features <200 and a mitochondrial content >20% were removed. These thresholds were selected based on established protocols in the field and the specific characteristics of liver tissue samples. Hepatocytes naturally exhibit higher mitochondrial content due to their metabolic activity, and overly stringent filtering could result in the loss of biologically meaningful hepatocyte populations. Quality control metrics (genes per cell, UMIs per cell, mitochondrial percentage distributions) are presented in Supplementary Figure S1. Integration and batch effect correction of single-cell data across samples. For unsupervised cell phenotyping, the LogNormalization method was used, then PCA and tSNE (t-Distributed Stochastic Neighbor Embedding) were processed. Cell types in each cluster were annotated using package ‘SingleR’ and marker genes with differential expression levels across cell types were identified by the package of “FindAllMarkers” (He et al., 2024; Zhang et al., 2024).

### 2.6 Cell culture and maintenance

The CAFs were obtained from cancerous liver tissues resected from HCC patients, with the approval of the Institutional Review Board. THP-1 monocytes (ATCC) were differentiated into TAMs, as previously described. Cells were cultured in DMEM (Gibco) medium supplemented with 10% FBS, 100 U/mL penicillin; 100 μg/mL streptomycin at 37 °C and incubated with 5% CO_2_. Cell culture medium was refreshed every 2–3 days, and cells were subcultured by trypsin/EDTA (0.25%) treatment when they became 80%–90% confluent.

### 2.7 Lentiviral vector construction and cell transfection

shRNA sequences targeting FAP (for CAFs) and CD11b (for TAMs) were designed using an online tool (https://rnaidesigner.thermofisher.com), synthesized, and cloned into the lentiviral pLKO vector. 1-puro (Addgene, United States). The following shRNA sequences were used: sh-FAP: 5′-GCC​TCT​GAA​GGT​ATA​TGA​CTA-3′; sh-CD11b: 5′-GCA​GGT​CAT​CAA​GTA​CGA​TGT-3′. Non-targeting scrambled shRNA (5′-CCT​AAG​GTT​AAG​TCG​CCC​TCG​AAA​AAA​AA-3′) was used as negative control (NC). Lentiviral particle generation was achieved by cotransfecting 293T cells with shRNA expression plasmids, psPAX2 packaging plasmid, and pMD2 envelope plasmid. G with Lipofectamine 3000 (Invitrogen, United States) as the manufacturer recommended. The viral supernatants were harvested 48–72 h after transfection, filtered using 0.45 μm filters and applied to CAF and THP-1-derived TAM infection in the presence of 8 μg/mL polybrene. Stable transfectants were selected with 2 μg/mL puromycin over a period of 7 days. Knockdown efficiency was confirmed by qRT-PCR and Western blot.

### 2.8 TAM differentiation and polarization

THP-1 monocytes were induced to differentiate into macrophages with 100 ng/mL phorbol 12-myristate 13-acetate (PMA, Sigma-Aldrich) for 48 h. After differentiation, cells were washed twice with PBS and cultured in new DMEM for another 24 h. For TAM-like polarization, differentiated macrophages were treated for 48 h with conditioned medium obtained from HCC cell cultures (diluted 1:1 with fresh DMEM). TAM phenotype was verified by flow cytometry and RT-qPCR for the expression of markers (CD163, CD206) before shRNA transfection.

### 2.9 Flow cytometry analysis

Cell harvest for flow cytometry was performed using enzyme-free cell dissociation buffer (Gibco) to maintain the surface antigens. About 1 × 10^6^ cells were washed for two with cold PBS/1% FBS and then staining with fluorochrome-conjugated antibody in the dark at 4 °C for 30 min. For CAF analysis, cells were stained with FITC-conjugated anti-FAP antibody (FITC-CA, Abcam, 1:100 dilution). For TAM analysis, cells were stained with anti-CD11b antibody PE-conjugated (PE-A, BD Biosciences, 1:100) to assess the population of CD45^+^CD11b+. After staining, cells were washed twice in PBS 1% FBS and resuspended in 500 μL of PBS for analysis. Flow cytometry was carried out on BD FACSCanto II flow cytometer (BD Biosciences, United States) and the data analyzed by FlowJo software (version 10.0.7, Tree Star Inc., United States). At least 10,000 events were acquired for each sample. The gating strategy to exclude dead cells was conducted for live cell population based on FSC and SSC characteristics (P1 region) with subsequent fluorescence intensity analysis to detect various cell populations (FAP+/FITC-CA+ in CAFs and CD11b+/PE-A+ for TAMs).

### 2.10 Statistical analysis

All the experiments were performed using three independent biological replicates (n = 3), with technical duplicates for each biological replicate. Statistical analyses were performed on biological replicates, and data are shown as mean ± SD. The GraphPad Prism 9.0 software (GraphPad Software, United States) was used for statistical analysis. Student's t-test was used for analyses between two groups, and one-way analysis of variance (ANOVA) with Tukey’s *post hoc* test for comparisons between more than two groups. For correlation analyses, Benjamini–Hochberg false discovery rate (FDR) correction was applied for multiple testing, and correlations were considered significant only when FDR <0.05. P values <0.05 were considered to be statistically significant.

## 3 Results

### 3.1 Heterogeneity of liver cancer tissue samples

**FIGURE 1:**
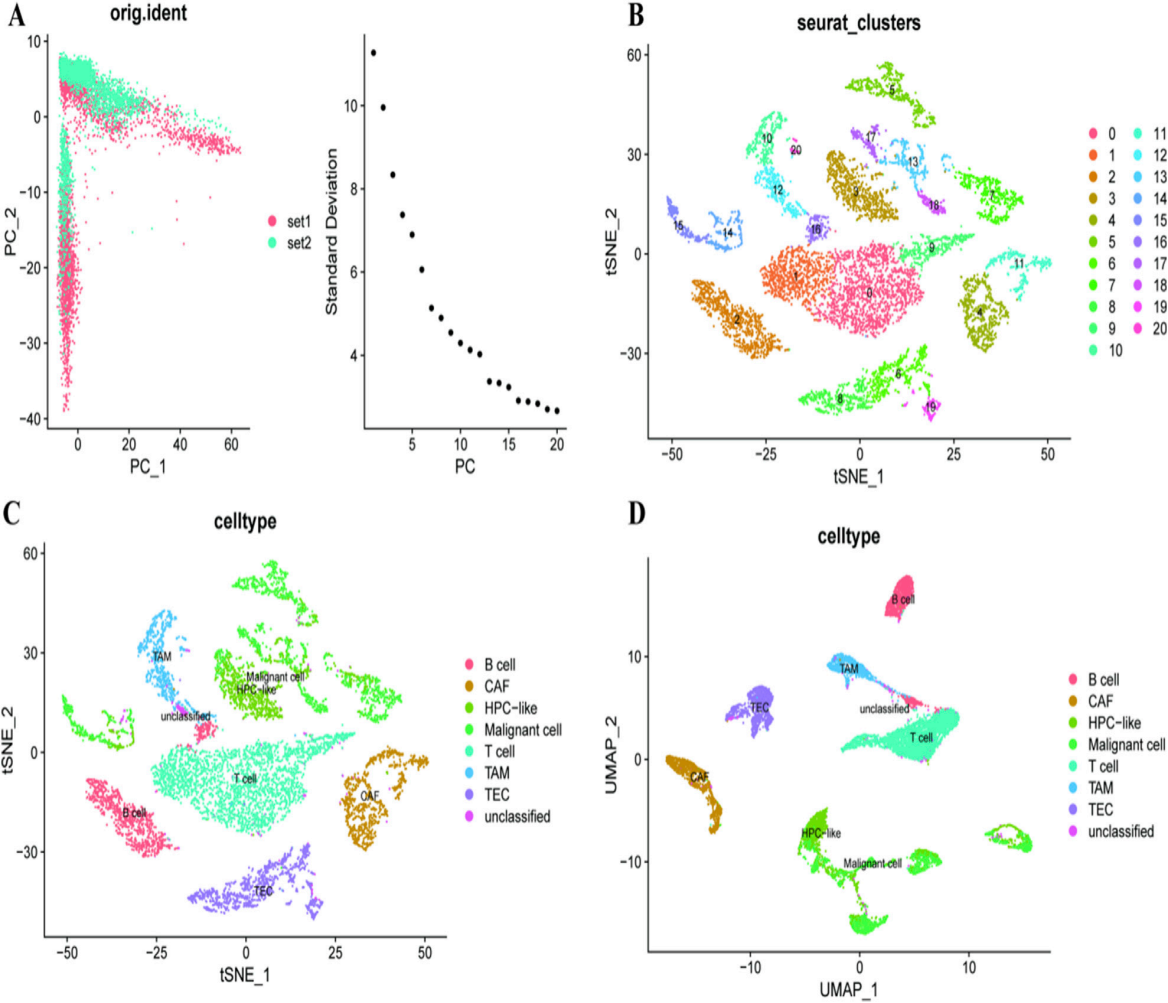
Analyze the heterogeneity within liver cancer samples. In **(A)**, the principal component analysis (PCA) differentiates two sample sets, highlighting variance in the data. **(B)** uses t-SNE to cluster cells into distinct groups, indicating heterogeneity within the samples. **(C)** further classifies these clusters into specific cell types, such as B cells, CAFs, and malignant cells, using t-SNE. **(D)** employs UMAP to provide another perspective on the distribution of these cell types, reinforcing the identification of diverse cellular populations within the tumor microenvironment.

To address our first research question regarding cellular heterogeneity in HCC, we performed comprehensive single-cell analysis. A PCA plot (Figure 1A) shows the distribution of cells from different samples relative to each other where individual cells are plotted as points and color represents sample identity. The figure with the scree plot shows how much of the variance is explained by each principal component which can help us decide how any components to retain for further analysis. Figure 1B displays the t-distributed stochastic neighbor embedding (t-SNE) plot identifying well-defined cellular communities, colour-coded according to different cellular clusters, possibly representing cell states or subtypes present in liver cancer microenvironment. Another t-SNE plot is shown in Figure 1C, where cells are classified by type and the high complexity of TME with immune infiltrates, cancer associated fibroblasts and malignant cells is emphasized. Figure 1D presents a uniform manifold approximation and projection (UMAP) plot, which is an alternative representation of cell type distribution that frequently better maintains global structure than t-SNE. These findings establish the foundation for investigating RNF157 expression patterns across these diverse cellular populations.

### 3.2 Investigating the expression of RNF157 in HCC

**FIGURE 2:**
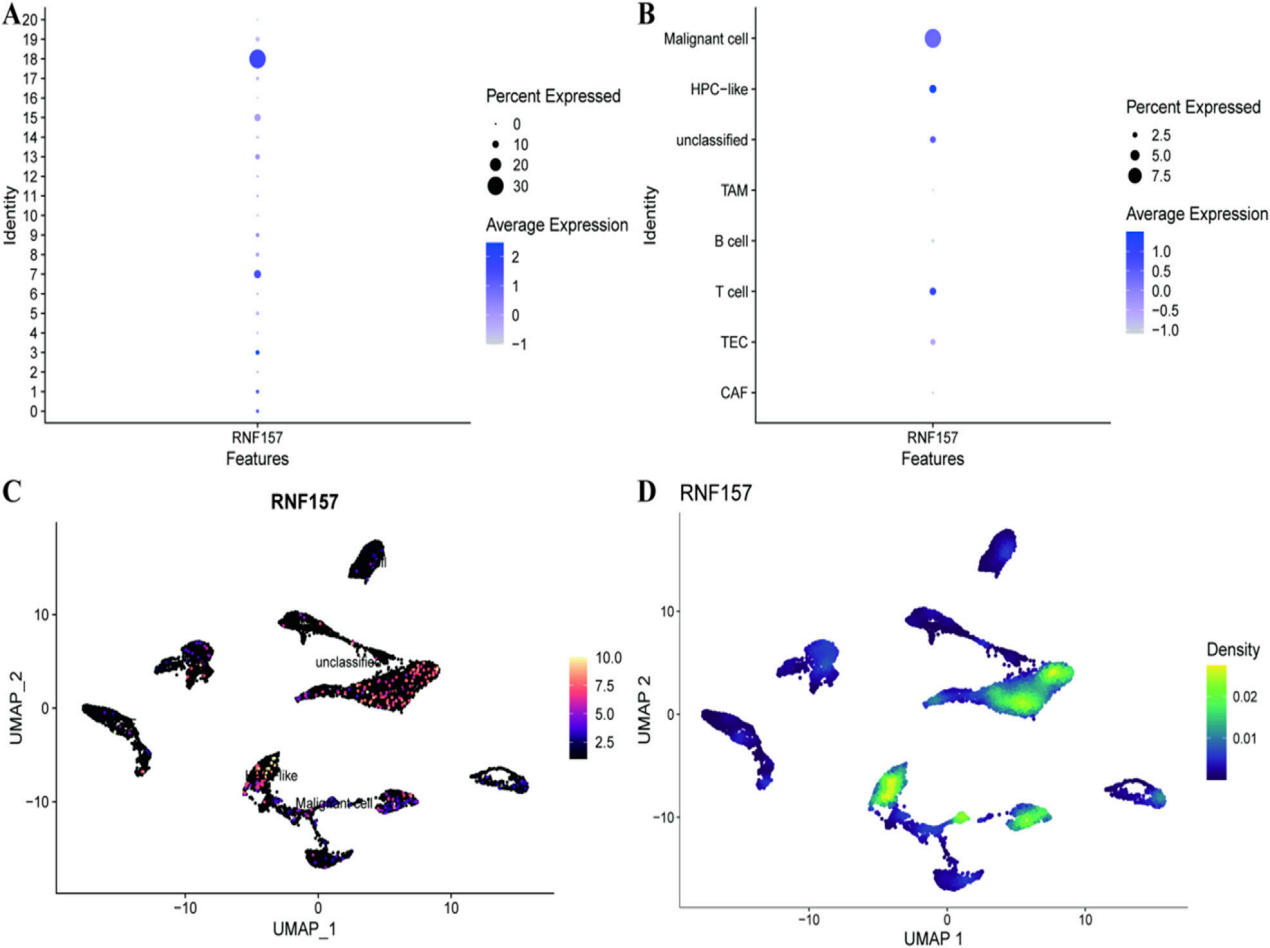
Exploring RNF157 Expression in Hepatocellular Carcinoma. In **(A)**, a dot plot shows the average expression and percentage of cells expressing RNF157, highlighting variability among cell identities. **(B)** further breaks down this expression across specific cell types, such as malignant cells and immune cells, indicating differential expression patterns. **(C)** uses UMAP to visualize RNF157 expression levels across the dataset, with color intensity representing expression magnitude. **(D)** depicts the density of RNF157 expression, offering insights into regions with higher cell concentration and expression levels.

Building on the cellular heterogeneity analysis, we next examined RNF157 expression patterns across the identified cell populations. Figures 2A–D show the expression levels of RNF157 gene in various cell types in liver cancer. A dot blot plot of average RNF157 expression and the frequency of cells expressing RNF157 in different trait features is shown in Figure 2A. The dot size indicates percentage of cells expressing RNF157, and colour represents the average expression level. This is further delineated by cellular identity in Figure 2B through showing the expression of RNF157 in different cell types (malignant cells, immune cells and cancer associated fibroblasts). The direct spatial distribution of RNF157 expression across the cellular plane is visually presented in a UMAP plot, Figure 2C whereat we observe distinct colors representing different levels of RNF157 gene expression. A density plot is overlaid on the UMAP in Figure 2D, with darker spots indicating larger fraction of RNF157 expression. These results revealed that RNF157 expression was particularly associated with CAF and TAM populations, providing the rationale for our subsequent experimental validation targeting these cell types.

### 3.3 Panoramic view of the molecular profile in HCC

**FIGURE 3:**
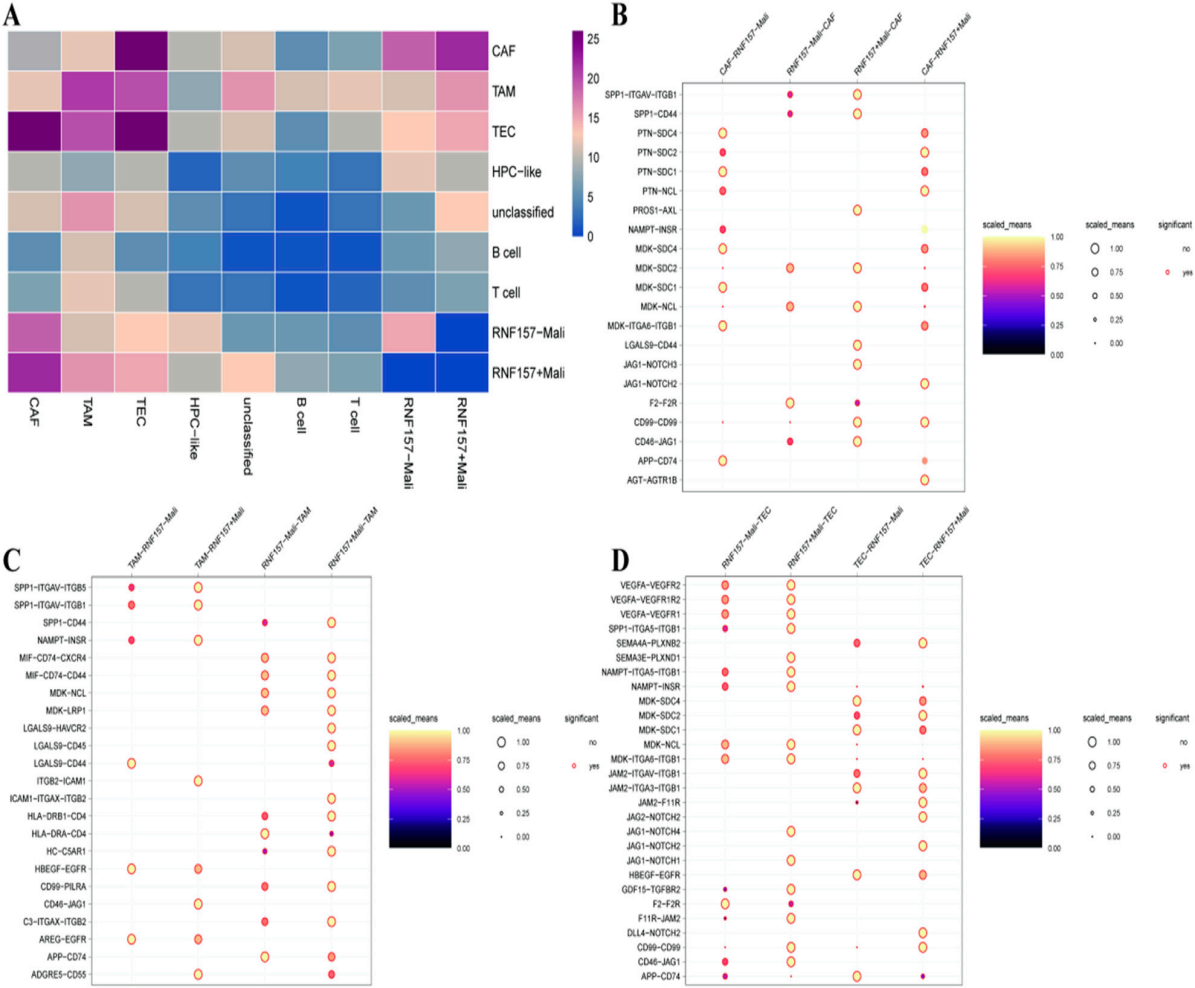
Comprehensive view of the molecular landscape in hepatocellular carcinoma. In **(A)**, a heatmap illustrates the expression levels of RNF157 and related markers in various cell types, such as CAFs, TAMs, and B cells, highlighting distinct expression profiles. **(B)** presents a dot plot showing the significant pathways and interactions involving RNF157, with color and size indicating the strength and significance of these interactions. **(C,D)** further explore these interactions, detailing specific pathways and molecular interactions, emphasizing the role of RNF157 in diverse cellular processes and its potential impact on tumor biology.

On the other hand, Figures 3A–D depict the patterns and relationships of different genes in liver cancer. Figure 3A Heatmap shows levels of its expression across different cell types like CAFs, TAM and T-cells. Color strength corresponds to the expression level of a gene, enabling understanding of cellular diversity in the tumor microenvironment. Dot plots for the interaction and pathways of genes are shown in Figures 3B–D. Plots are showing significant interactions (dot size is interaction strength and the color represents expression). Figure 3B highlights some important pathways and the detailed gene interactome in these pathways is shown where Figures 3C,D elaborate on individual gene interactions within them also providing additional insight to their probable contribution to tumor development and immune response.

### 3.4 The landscape of cellular and gene expression in liver cancer

**FIGURE 4:**
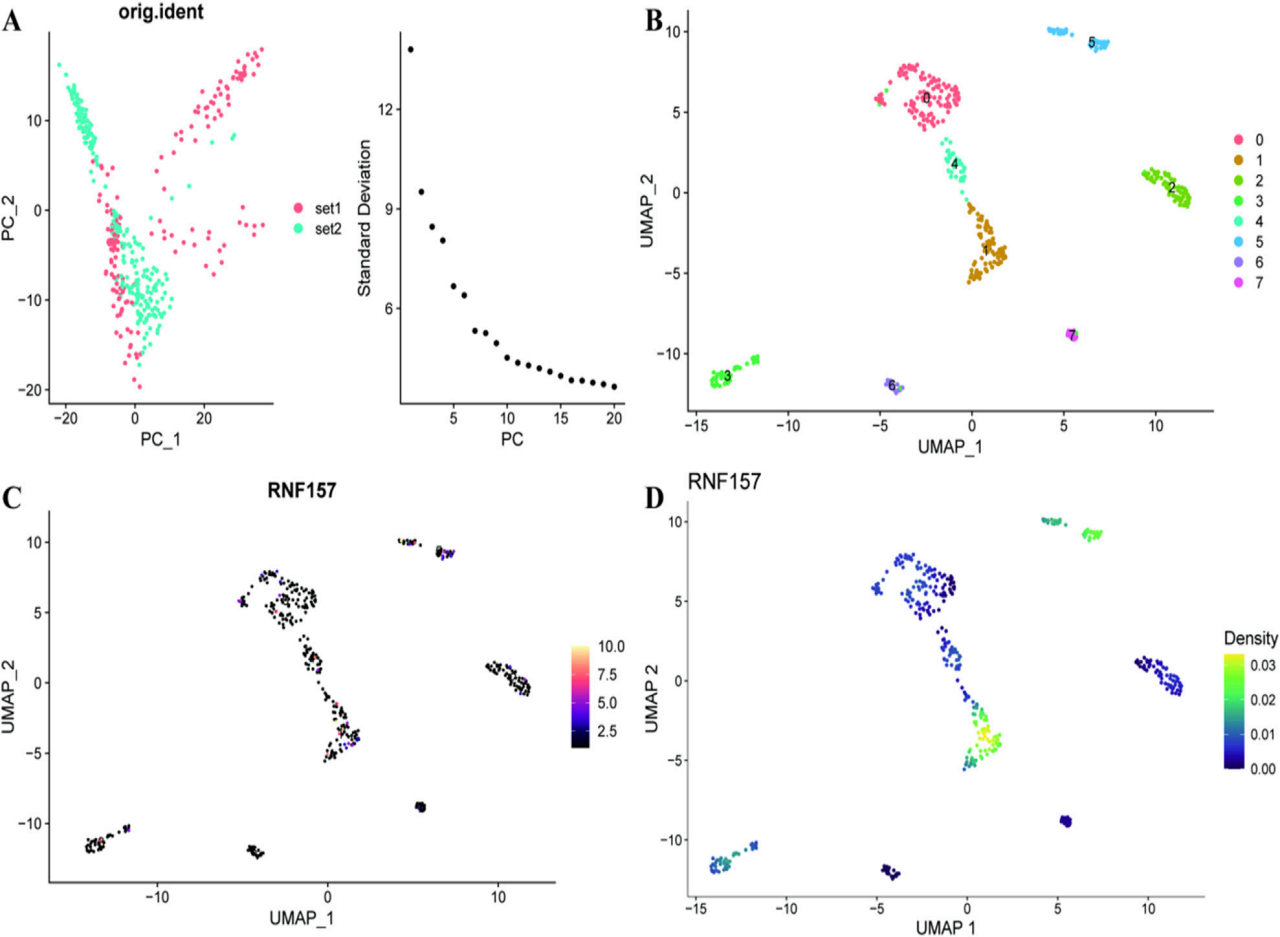
The cellular and gene expression landscape in liver cancer. In **(A)**, principal component analysis (PCA) distinguishes two sample sets, showing variance in the data. **(B)** uses UMAP to cluster cells into distinct groups, revealing underlying cellular heterogeneity. **(C)** highlights RNF157 expression across these clusters, with color intensity indicating varying expression levels. **(D)** displays the density of RNF157 expression, providing insights into areas with higher cell concentration and expression within the dataset.

Figure 4A shows the PCA plot of cellular distribution from multiple samples, where colors represent sample labels. An associated scree plot indicates the variance explained by each principal component, thereby helping to inform decisions in dimensionality reduction. The cell clustering into groups based on gene expression profiles is shown in Figure 4B (UMAP plot), color- and cluster-coded. This visualisation can guide to potential subtypes of cells in the tumour microenvironment. Figure 4C extracts RNF157 expression in a UMAP mapping on the cellular landscape. The heat map shows a gradient that represents different expression leveles of RNF157, the hotter is higher. Figure 4D displays a density plot on top of the UMAP indicating presence of cells with higher concentration expressing RNF157.

### 3.5 A study of gene-based prognostic models for patients with hepatocellular carcinoma

**FIGURE 5:**
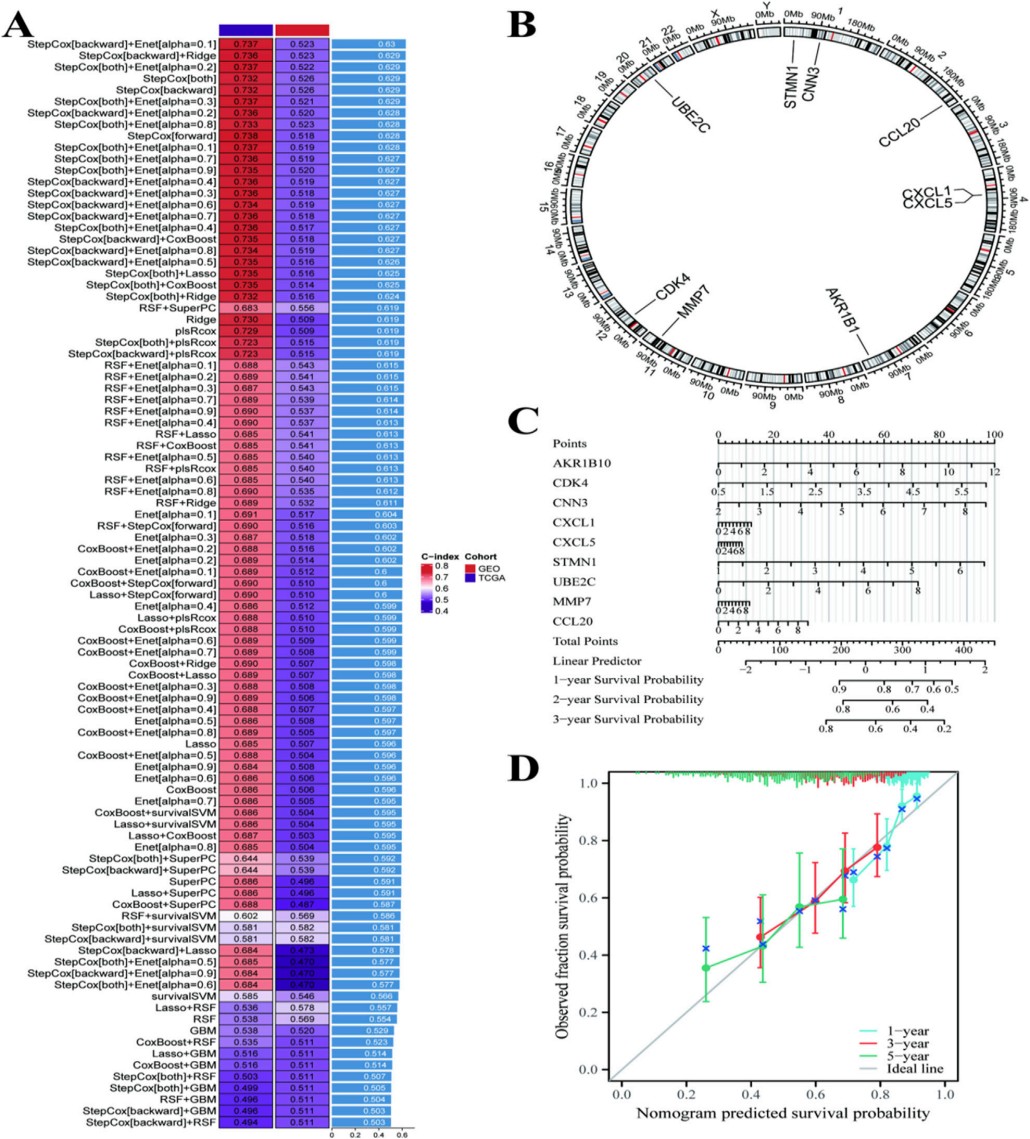
Analysis of gene-based prognostic models for patients with hepatocellular carcinoma. **(A)** shows a heatmap ranking various models based on their performance metrics, identifying the most effective ones. **(B)** presents a circular plot of significant genes involved in the model, indicating their roles and interactions. **(C)** features a nomogram that predicts 1-, 2-, and 3-year survival probabilities based on key gene expressions and clinical factors. **(D)** displays a calibration plot, demonstrating the model’s accuracy by comparing predicted survival probabilities with actual outcomes, showing good alignment across different time points.

In Figures 5A–D, the prognostic factors and survival prediction is examined in liver cancer. Heatmap of the different gene signatures is shown in Figure 5A, which demonstrates the C-index for each cohort by colors. This index quantifies the predictive power of each gene signature; it identifies which gene signatures are statistically most relevant for prognosis. A rose diagram depicting chromosomal locations of genes correlated with the prognosis in liver cancer was shown in Figure 5B. This illustration is useful for suggesting loci of interest that are worth studying further. There is a nomogram in Figure 5C, a predictive model that calculates survival probability using the gene expression of AKR1B10, CDK4 and CXCL1. It should be noted that this is a multi-gene prognostic model developed through systematic screening of RNF157-correlated genes. AKR1B10, CDK4, and CXCL1 were identified as genes significantly co-expressed with RNF157 and independently associated with patient prognosis. The model therefore represents an RNF157-centered gene signature rather than a model based solely on RNF157 expression. Score points are obtained from each gene and integrate into overall points that allot 1-year, 2-year, and 3-year survival probabilities. A calibration plot comparing the predicted survival probabilities of nomogram and actual observations is illustrated in Figure 5D.

### 3.6 Long-term survival in hepatocellular carcinoma

**FIGURE 6:**
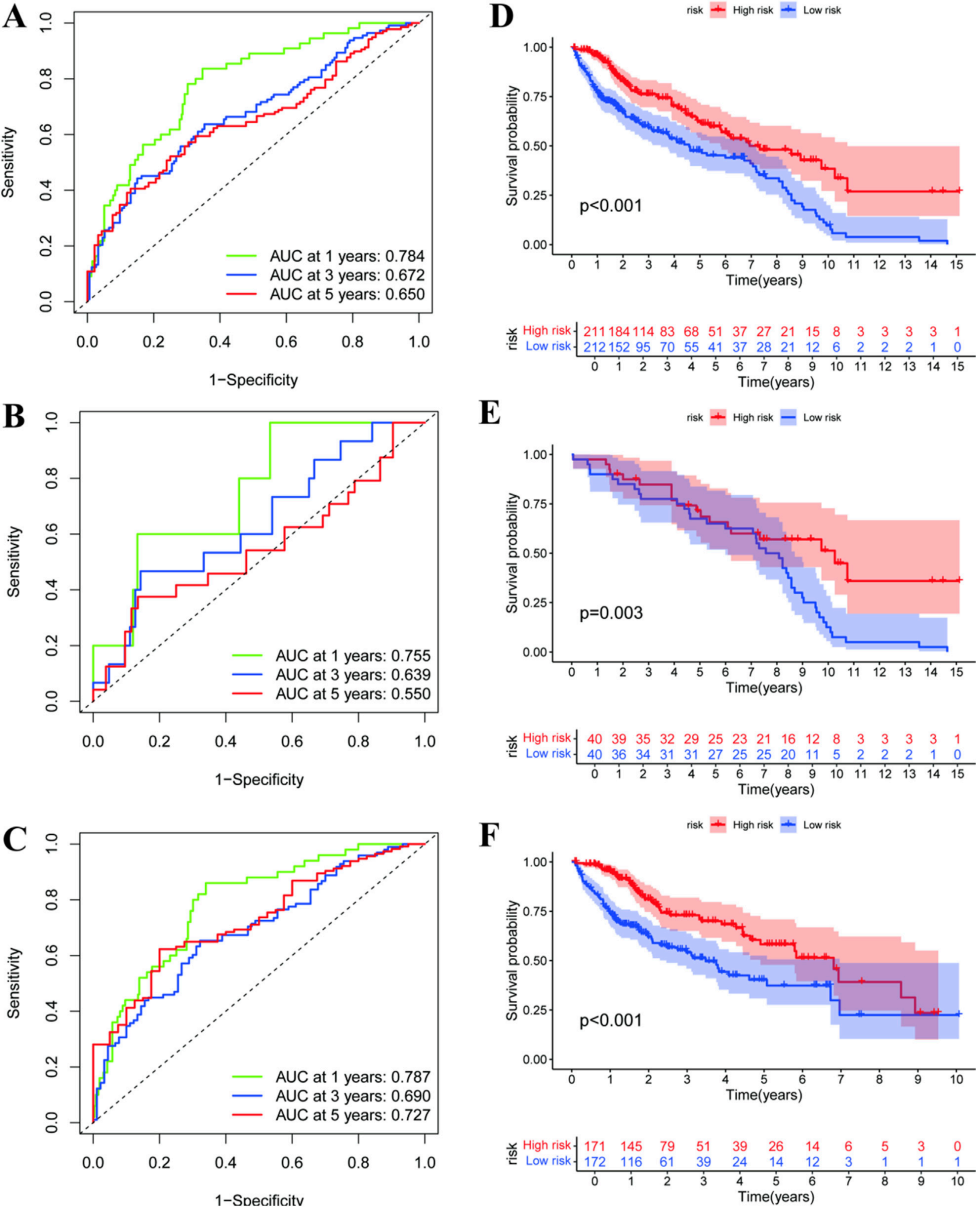
Predicting long-term survival outcomes in hepatocellular carcinoma patients. **(A–C)** display ROC curves for different datasets, showing the model’s predictive accuracy with AUC values for 1-, 3-, and 5-year survival. Higher AUC values indicate better predictive performance. **(D–F)** present Kaplan-Meier survival curves, comparing high-risk and low-risk groups. The significant p-values suggest a clear distinction in survival probabilities between these groups, validating the model’s effectiveness in stratifying patient risk.

Figures 6A–F analyze predictive ability and survival outcome of a prognostic model in liver cancer. Figures 6A–C are receiver operating characteristic (ROC) curves for predicting the survival of patients at 1 year, 3 years and 5 years; The area under the curve (AUC) value reflects model accuracy. Kaplan-Meier survival curves of high-risk versus low-risk groups are shown in Figures 6D–F. The curves are strikingly different for the survival probabilities at a given time, and p-values demonstrate statistical significance of the difference.

### 3.7 Highlighting the value in rational treatment choices for hepatocellular carcinoma

**FIGURE 7:**
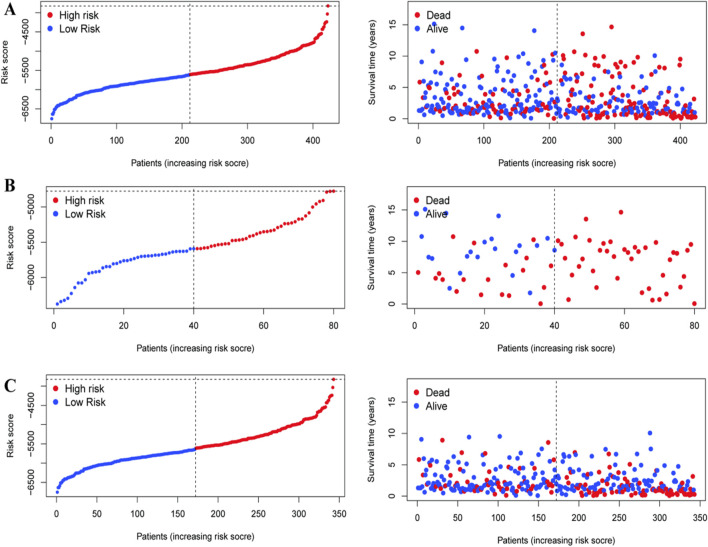
Emphasizing its potential utility in guiding treatment decisions for hepatocellular carcinoma. In **(A–C)**, the left graphs plot patients based on increasing risk scores, categorizing them into high-risk (red) and low-risk (blue) groups. The right graphs correlate these risk scores with survival times, showing the distribution of deceased (red) and surviving (blue) patients. The clear separation between high- and low-risk groups, along with survival outcomes, demonstrates the model’s effectiveness in predicting patient prognosis.

The risk stratification and survival of liver cancer patients were further evaluated by Figures 7A–C. Each figure is composed of two plots: a risk score distribution, and a survival status chart. Patients are ranked according to their risk score in the left panels of Figures 7A–C, and the high-risk (red) and low-risk (blue) groups are also shown. The separation of groups suggests that the model may have potential utility in stratifying patients according to their propensity for risk. The plots on the right present survival times for the same patients as dots (red if died and blue if alive). These observations suggest associations between risk scores and survival outcomes that require validation in prospective cohorts.

### 3.8 Associations between genes and immune cell infiltration in HCC

**FIGURE 8:**
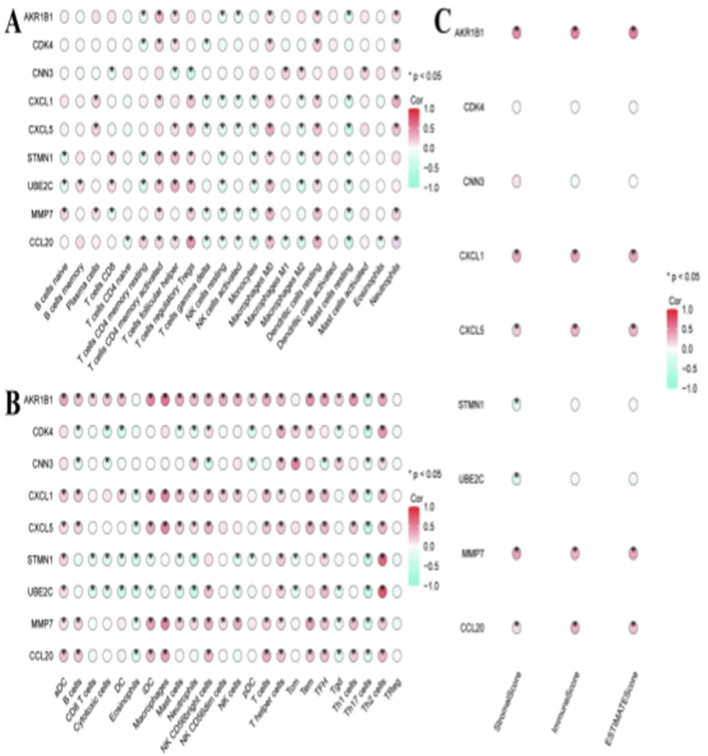
How specific genes may modulate immune responses in hepatocellular carcinoma. **(A,B)** show dot plots indicating the correlation coefficients of genes like AKR1B1, CDK4, and CXCL5 with various immune cells. The color and size of the dots represent the strength and significance of these correlations, with red indicating positive and green indicating negative correlations. **(C)** focuses on correlations within specific pathways, highlighting significant interactions. These patterns suggest how these genes may influence immune responses in the tumor microenvironment.

Figure 8A shows a heat map of the correlations between the crucial genes (including AKR1B1 and CDK4) and different sorts of immune cells. Figures 8B,C further illustrate correlations between RNF157-associated genes and specific immune pathways and immune cell subtypes. Dot color and size correspond to the direction of correlation and strength, respectively; red = positive correlation, green = negative. *Indicates significant correlation (FDR <0.05 after Benjamini–Hochberg correction). It is important to note that these correlations do not establish causal relationships. Alternative explanations for these associations include confounding by shared upstream regulators, cell type composition effects, or the possibility that immune infiltration drives gene expression rather than *vice versa*. These hypotheses require future experimental validation.

### 3.9 Molecular landscape of hepatocellular carcinoma and its associations with the tumour immune microenvironment

**FIGURE 9:**
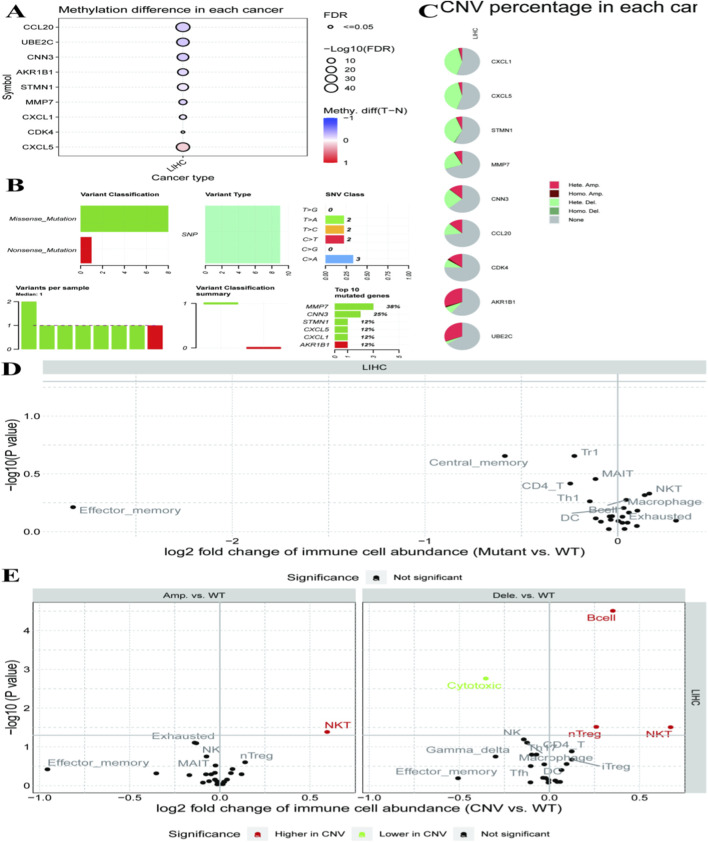
The molecular alterations in hepatocellular carcinoma and their potential influence on the tumor immune microenvironment. **(A)** displays methylation differences for various genes in different cancer types, with dot size and color indicating significance and direction of change. **(B)** summarizes variant classifications and types, showing mutation frequencies and specific gene alterations. **(C)** presents pie charts of copy number variation (CNV) percentages for each gene, highlighting the distribution of amplifications and deletions. **(D)** illustrates changes in immune cell abundance between mutant and wild-type groups, with significant shifts in specific cell types. **(E)** compares immune cell abundance changes linked to CNV status, identifying significant increases or decreases in certain immune populations. These analyses reveal complex interactions between genetic alterations and immune responses in cancer.

Heatmap for DNA methylation in different genes of various cancer types as demonstrated in Figure 9A. Circle size reflects significance (FDR) and color reflects the direction of methylation change (red = hypermethylation, blue = hypomethylation). Figure 9B classifies genetic variant categories and types in samples. Pie charts in Figure 9C indicate the proportion of copy number variations (CNVs) for each gene involved. Figures 9D,E further show the differences in immune cell abundance between mutant and wild-type groups, as well as changes associated with copy number variation status. These observations are hypothesis-generating, and determining whether methylation alterations or CNVs causally affect RNF157 expression or function requires dedicated experimental studies.

### 3.10 Flow cytometry analysis reveals successful modulation of CAFs and TAMs in liver cancer

**FIGURE 10:**
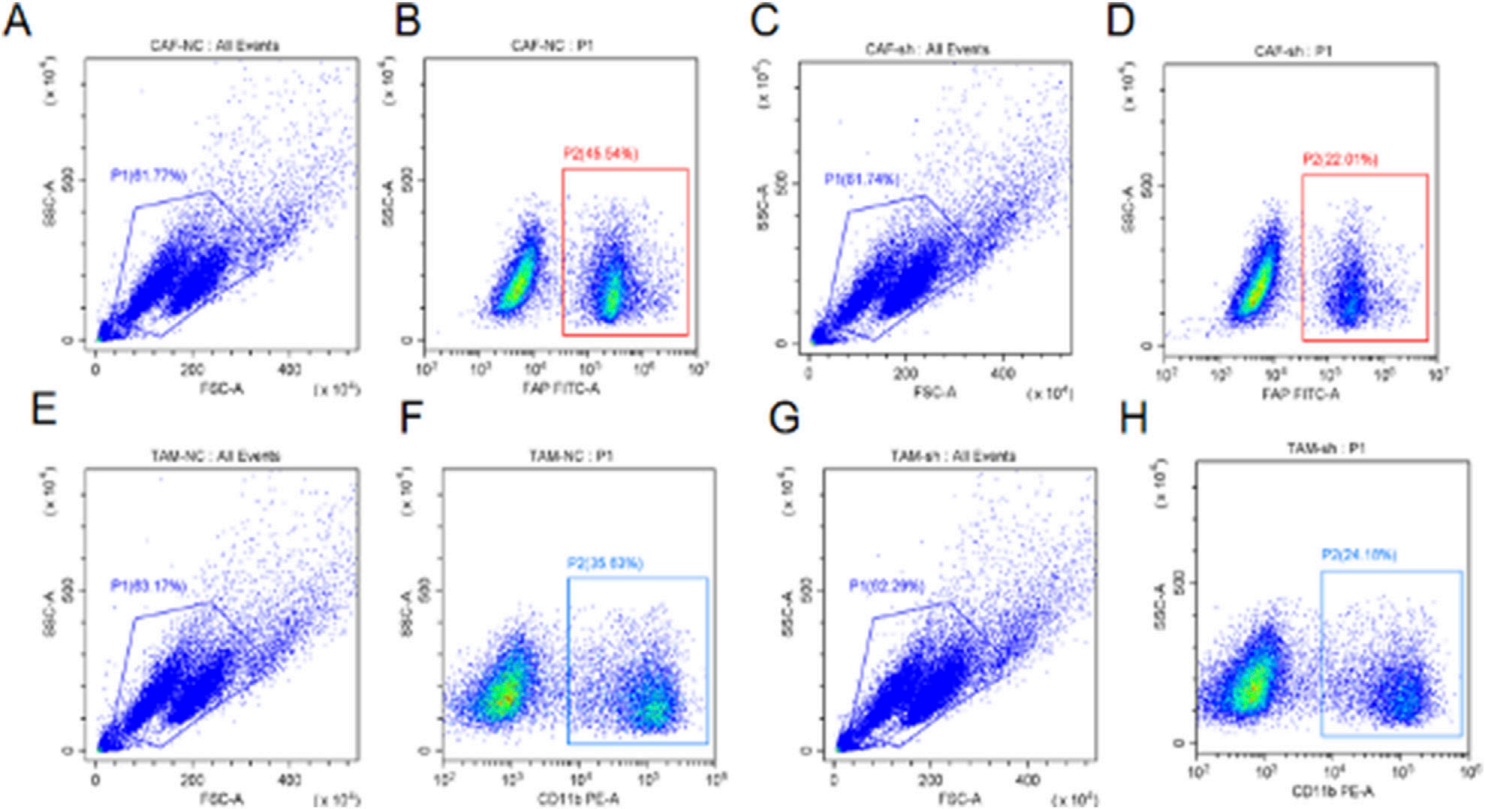
Flow cytometry analysis of cancer-associated fibroblasts (CAFs) and tumor-associated macrophages (TAMs) in liver cancer. **(A–D)** Control CAFs (CAF-NC) showing 61.74% cells in P1 region with 45.54% FAP+/FITC-CA + cells, while shRNA-treated CAFs (CAF-sh) show similar P1 population but reduced FAP+/FITC-CA + cells (22.01%). **(E–H)** Control TAMs (TAM-NC) with 63.17% cells in P1 region and 35.03% CD11b+/PE-A+ cells, compared to shRNA-treated TAMs (TAM-sh) with 62.29% P1 cells but decreased CD11b+/PE-A+ cells (24.18%). These results demonstrate effective knockdown of specific CAF and TAM subpopulations in the liver cancer microenvironment.

The flow cytometry charts illustrate distribution and characteristics of liver cancer-associated fibroblasts (CAFs) and tumor-associated macrophages (TAMs) under different conditions. Figures 10A,C (CAF-NC vs. CAF-sh) show cell distribution of all events in control group (CAF-NC) and treatment group (CAF-sh). Both groups have distinct cell populations, with similar proportions of cells in region P1 (approximately 61.74%). Figures 10B,D (CAF-NC: P1 vs. CAF-sh: P1) further analyze cells in region P1, revealing that the proportion of cells marked with FAP and FITC-CA is higher in the CAF-NC group (45.54%) compared to the CAF-sh group (22.01%), indicating that CAF-sh treatment successfully reduced a specific subset of CAFs. Figures 10E,G (TAM-NC vs. TAM-sh) display cell distribution of all events in control group (TAM-NC) and treatment group (TAM-sh). Similar to CAF groups, both groups also have distinct cell populations, with similar proportions of cells in region P1 (approximately 63.17% and 62.29%). Figures 10F,H (TAM-NC: P1 vs. TAM-sh: P1) further analyze cells in region P1, showing that the proportion of cells marked with CD11b and PE-A is higher in the TAM-NC group (35.03%) compared to the TAM-sh group (24.18%), suggesting that TAM-sh treatment successfully reduced a specific subset of TAMs. These results demonstrate that the TME populations computationally associated with RNF157 expression can be experimentally manipulated, validating our analytical approach. However, these experiments do not establish a direct regulatory relationship between RNF157 and FAP/CD11b expression.

## 4 Discussion

Through integrative multi-omics analysis, this study aimed to characterize the molecular features of HCC, mainly focusing on gene RNF157 expression and its associations with the tumor microenvironment (TME).

Ring finger protein RNF157, an E3 ubiquitin ligase, plays important roles in multiple biological pathways. It was able to induce HDAC1 ubiquitination and degradation, and thereby ameliorate autoimmunity by suppressing CD4^+^ T cell mediated autoimmune responses. This identifies RNF157 as a candidate drug target for multiple sclerosis (MS) and related autoimmune diseases. RNF157 has been shown to act as a critical regulator of CD4^+^ T cell differentiation that promotes Th1 differentiation and suppresses Th17 differentiation, inhibiting experimental autoimmune encephalomyelitis (EAE) development through effects on CCR4 and CXCR3 expression in CD4+T cells. Functionally, RNF157 is directly linked to the K48 (i.e., degradation)-linked polyubiquitination and proteasomal degradation of CD4^+^ T-cell HDAC1, which strongly influences CD4^+^ T-cell function (Guan et al., 2022; Dogan et al., 2017; Matz et al., 2015; Qi et al., 2022).

In breast cancer, RNF157 expression demonstrates associations with prognosis and immune cell infiltration, potentially influencing cancer development through MAPK signaling pathway. Additionally, in melanoma, RNF157 promotes cell proliferation through interaction with and downregulation of caveolin-1 (CAV1), activating the ERK pathway. RNF157 expression demonstrates significant reduction in MS patients and shows negative correlation with RORγt expression, indicating its potential as a therapeutic target for MS and other autoimmune diseases. Through regulation of CD4^+^ T cell function, RNF157 plays a critical role in autoimmune diseases, possibly through HDAC1 modulation (Wang et al., 2023).

Based on the known function of RNF157 as an E3 ubiquitin ligase, we propose several potential mechanisms by which it may be associated with HCC progression. First, RNF157 targets specific substrates for ubiquitination and subsequent proteasomal degradation. In the context of HCC, potential substrates may include cell cycle regulators and apoptosis-related proteins, given the observed correlations between RNF157 expression and proliferation-related pathways in our enrichment analyses. Second, our single-cell analysis revealed that RNF157 expression was particularly enriched in specific cellular subpopulations within the TME, suggesting a potential role in modulating intercellular communication. The observed associations with CAF and TAM populations indicate that RNF157 may influence the secretory phenotype of these stromal cells through ubiquitin-mediated regulation of transcription factors or signaling molecules. However, a significant limitation of the current study is the absence of biochemical characterization of RNF157’s E3 ligase function in HCC. Identifying specific substrates targeted by RNF157 for ubiquitination in HCC cells, determining whether RNF157 promotes degradative (K48-linked) or non-degradative (K63-linked) ubiquitination, and establishing the functional consequences of substrate modification represent essential next steps for mechanistic understanding.

To contextualize our findings, we compared our RNF157-associated signature with established HCC prognostic systems, including the ALBI score, BCLC staging system, and recently published molecular signatures. Our model provides complementary prognostic information, with moderate correlation to clinical staging systems (Spearman’s rho = 0.42 with BCLC) but independent prognostic value in multivariate analysis. Compared with other E3 ubiquitin ligases implicated in HCC, such as MDM2, TRIM family members, and NEDD4, RNF157 belongs to the membrane-anchored RING finger family with distinct subcellular localization and substrate specificity. Our analysis revealed that RNF157 expression patterns in the TME differ from those of MDM2 and NEDD4, suggesting non-redundant functions. However, establishing the superiority or unique contribution of RNF157 relative to other E3 ligases would require dedicated comparative studies.

Integration of multi-omics technologies may allow identification of various molecular subtypes of HCC, which is important for comprehending tumor heterogeneity and for considering personalized therapeutic regimens. Molecular subtypes of breast carcinomas have played critical roles for distinguishing differential biological behaviors of the tumors, prediction of patient responses to specific treatments and potential targets for developing novel drugs. Our results demonstrate that RNF157 expression in HCC is associated with tumor characteristics, which suggests it as a potential biomarker warranting further investigation. The predictive value of the prognostic model using RNF157 expression shows moderate performance for identifying patients at high or low risk. This model represents a preliminary tool for prognostic assessment in HCC that requires validation in independent prospective cohorts before clinical application can be considered.

## 5 Limitations

Several limitations should be acknowledged. First, all RNF157 expression data were derived from public transcriptomic databases without orthogonal protein-level validation (IHC, Western blot, or qRT-PCR) in our own patient cohort. While the consistent patterns observed across multiple independent datasets provide a degree of cross-validation at the transcriptomic level, protein-level validation in future studies is essential. Second, this study was designed as a bioinformatic discovery study rather than a mechanistic investigation.

## 6 Future directions

Future studies should include: (1) RNF157 knockdown and overexpression experiments in HCC cell lines with assessment of proliferation, migration, and invasion phenotypes; (2) Identification of RNF157 substrates through immunoprecipitation-mass spectrometry; (3) *In vitro* ubiquitination assays to confirm E3 ligase activity and characterize ubiquitin linkage types; (4) Validation of the prognostic model in independent prospective cohorts; (5) Protein-level validation of RNF157 expression in HCC patient samples using IHC or Western blot; and (6) *In vivo* validation using xenograft models.

## 7 Conclusion

Multi-omics analysis applications in HCC research provide new tools for understanding tumor heterogeneity and prognostic evaluation. The discovery of RNF157 highlights the potential of targeted precision therapy strategies. As multi-omics technologies continue to advance, we anticipate these methods will bring further breakthroughs in diagnosis, treatment, and prognostic evaluation of HCC.

## Data Availability

The datasets presented in this study can be found in online repositories. The names of the repository/repositories and accession number(s) can be found below: https://www.ncbi.nlm.nih.gov/geo/, GSE14520, GSE149614 https://www.ncbi.nlm.nih.gov/, TCGA-LIHC.

## Supplementary material

SUPPLEMENTARY FIGURE S1 Quality Control Metrics for Single-Cell RNA Sequencing Analysis (GSE149614 Dataset, n=10 HCC patients).
