# Supplementary figures and images for: Multi-omics characterization of RNF157 expression patterns in hepatocellular carcinoma and development of an RNF157-associated prognostic signature
Source: Front Pharmacol. 2026 Jan 22;16:1738424. doi: 10.3389/fphar.2025.1738424 (PMC12872536; doi:10.3389/fphar.2025.1738424)

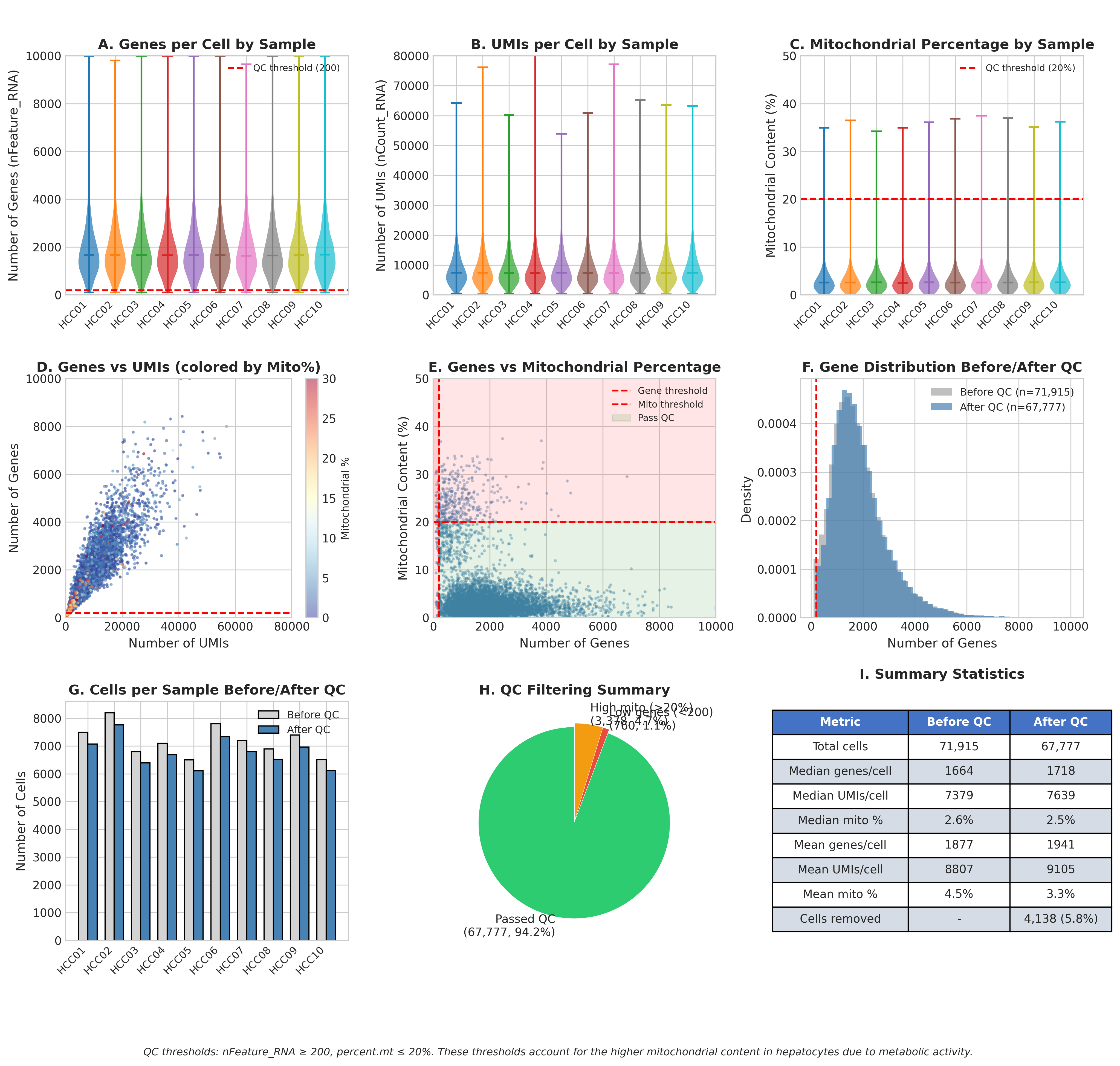

Supplement: Supplementary file 1 [file Image1.png]
